# Capacity development in patient-oriented research: programme evaluation and impact analysis

**DOI:** 10.1186/s12961-020-00606-9

**Published:** 2020-08-10

**Authors:** Melanie King Rosario, Marilynne A. Hebert, Balreen Kaur Sahota, Dean Eurich

**Affiliations:** 1 University of Calgary, 2500 University Dr. NW, Calgary, AB T2N 1N4 Canada; 2 University of Alberta, 116 St and 85 Ave., Edmonton, AB T6G 2R3 Canada

**Keywords:** Health research capacity development, Patient-oriented research, Programme evaluation, Health research trainee development, Impact analysis

## Abstract

**Background:**

National and provincial funding was invested to increase the quantity and quality of patient-oriented research (POR) across Canada. Capacity development became a priority to ensure all stakeholders were prepared to engage in POR. In part, this need was met through an annual Studentship competition in the province of Alberta, providing funding to students whose research incorporated principles of POR. However, despite efforts to build capacity in the health research trainee population, little is known about the outcomes of these programmes. This evaluation study examined the outcomes of a POR capacity development programme for health research trainees.

**Methods:**

Final impact narrative reports were submitted by the 21 Studentship programme awardees for 2015 and 2016 who represent a variety of health disciplines across three major research universities. The reports describe the programme outcomes as well as the overall impact on individual, project and professional development as POR trainees. A synthesis of structured and categorised report data was conducted, along with additional qualitative analyses as new themes emerged that were not apparent in the competency framework utilised in the programme design.

**Results:**

Awardee reports detailed the impact of the Studentship programme on the key themes of increased knowledge and skill, relationship building, confidence and leadership, as well as project and career impact. The impacts felt most profoundly by the awardees were not reflective of the competencies that guided programme design. The outcomes were then re-examined using a health research capacity development framework to gain a more comprehensive view of programme impact.

**Conclusion:**

The Studentship programme narratives provided insight into the rarely tracked capacity development outcomes of POR research trainees. Awardee narratives indicated significant development beyond the intended competencies and suggested a need to revisit the competency framework for POR in Alberta. While competencies were useful in guiding the design of the initial programme, a more comprehensive capacity development framework was required to capture the broader impacts on trainee development. Future capacity development programmes may benefit from these early programme insights, specifically the need for more robust competencies for POR. Further exploration of evaluation methods for short-term awards and sustainability of capacity development programmes is warranted.

## Plain English summary

Health research priorities have generally been decided by researchers and funding agencies. Patient-oriented research (POR) changes this approach and includes patients as partners in research. A national funder in Canada has invested in the Strategy for Patient-Oriented Research (SPOR) initiative that supports a change in this direction. As the new SPOR initiative was implemented across the country, the need for capacity development for all stakeholders, from researchers to patients, students, clinicians and the community at large, became evident. Health research trainees (graduate students in Master’s and PhD programmes) were a significant target audience for POR training due to their potential to embrace these new trends as they moved forward in their careers. Despite the variety of capacity development activities offered to graduate students in health research, little is known about the impact of these activities.

A multidisciplinary stakeholder team designed a Studentship programme that is aligned with joint national and provincial priorities in POR. Since 2014, successful applicants have been awarded a Studentship through a rigorous provincial competition. At the conclusion of each 1-year funding cycle, outcomes were assessed through awardee participation in a training programme as well as in narrative reports. These narratives demonstrated outcomes and impacts of the Studentship programme with respect to awardees’ POR knowledge, skills, confidence, leadership and career networking. The narratives also illustrated the effects on personal attitudes and perspectives as well as changes in awardees’ research projects, future goals and career trajectories.

A competency framework was utilised to develop the Studentship programme; however, the awardee outcomes were much broader than the competencies suggested. An established health research capacity development framework was utilised to examine the results through a new comprehensive lens that also included personal development and the greater research environment. This is the first study in Canada to examine the outcomes of POR capacity development activities for research trainees. It provides important insights into the use of national and provincial competencies for POR as well as an overall positive impact in investing in health research capacity development.

## Background

Patient and community collaboration in health research has become a growing global trend in an effort to improve health outcomes and services delivery as well as to ensure the relevance of research studies to end-users, including patients and families. Public involvement in health research has been described using a variety of terms and implemented through government and industry investment to improve the relevance and impact of health research around the world [1, 2]. The Canadian Institutes for Health Research (CIHR) responded to this need through the development and funding of the Strategy for Patient-Oriented Research (SPOR). The purpose of SPOR is to increase the quantity of patient-oriented research (POR) conducted in Canada and to provide support to ensure this research is of high quality [3].

POR is an approach to health research that values the collaboration of patients, family and the public as more than just subjects but also as experts in their healthcare experience, disease area and as key stakeholders in health research results. POR represents a culture change in the health research community in which patients no longer play a passive role and researchers are no longer the only experts at the table [4]. Patients are now positioned as part of the research team with a fundamental role in determining research priorities, developing research questions and other critical tasks in the research process [5].

The implementation of the SPOR initiative varies across Canada, through Support for People and Patient-Oriented Research and Trials (SUPPORT) Units. Provinces and regions across Canada have designed and implemented their own SPOR SUPPORT Units to advance POR in their health system [6]. This variation in SUPPORT Unit implementation allows for contextual relevance, with support and services tailored to the needs and population of the area. The Alberta SPOR SUPPORT Unit (AbSPORU) received matched funding from CIHR and Alberta Innovates. AbSPORU has expertise in various areas of POR and health research, including patient engagement, knowledge translation, health research methods, data science, pragmatic clinical trials, consultation services and capacity development. In collaboration with a variety of stakeholders and key audiences, the Capacity Development Platform focuses on building POR knowledge and skills and translating these into practice.

## Capacity development

Capacity development is the process of individual and institutional development of higher levels of skills and greater ability to perform useful tasks [7]. In health research, capacity development aims to improve the ability of individuals and teams conducting research as well as to increase the effectiveness of the results [8, 9]. CIHR describes the capacity development goals of SPOR as initiatives designed to ensure that all partners in health research are trained and prepared for the collaborative work of POR [3]. A similar initiative in the United States, the Patient-Centered Outcomes Research Institute (PCORI), suggested that traditional research approaches were often a barrier to true engagement with patients and recommended training and development as a key priority in addressing this gap [10].

Research trainees are considered a key audience for capacity development initiatives because they are at the forefront of new research and can play a role in the sustainability and advancement of fields like POR as they build their research careers [11]. Training early career researchers is a critical step in shaping the future of health research and ensuring that trainees reach their full potential [4]. As graduate work often sets the tone for a future career in health research, this population was targeted as a key factor in POR capacity development in Alberta.

Effective research capacity development programmes should include a continuum of activities to meet various needs and support projects [12]. Additionally, they should contribute to a strong research culture and build the research workforce, particularly among new and emerging researchers [13]. Activities commonly include education, mentorship, funding, partnerships and networking [13–16].

National POR competencies were developed to guide the design of capacity development activities in an effort to provide consistency in the programmes offered by SPOR and provincial SUPPORT Units [17]. The national competencies were developed as a curriculum co-designed with patient partners as part of the SPOR strategy [18]. Bell et al. [19] acknowledged the tension in implementing the national curriculum and adapting the content to local contexts. In Alberta, the competencies were used to guide local programmes with additional competencies developed to account for areas of focus in AbSPORU. These introductory competencies were used to design and develop the Studentship programme (Table 1).

**Table 1:** Combined Canadian Institutes for Health Research and AbSPORU competency themes for POR

POR competency themes^a^	National SPOR capacity development competencies	Additional Alberta AbSPORU capacity development competencies
Patient-oriented research	✔	
Patient engagement	✔	✔
Health research	✔	
Communications & collaboration	✔	
Health research methods		✔
Knowledge translation & knowledge synthesis		✔
Data		✔
Clinical trials		✔

*AbSPORU* Alberta SPOR SUPPORT Unit, *POR* patient-oriented research, *SPOR* Strategy for Patient-Oriented Research

^a^Each competency theme is further divided into specific competencies and/or learning objectives for that theme area

## Programme description and rationale

In 2015, stakeholders (including students, patients, researchers, educators and administrators) participated in a working session to develop the vision and framework for the Studentship programme. The resulting priorities included patient involvement in learning activities, mentorship and networking with all stakeholders as well as opportunities to be involved in the broader POR and research communities. Given this direction as well as the POR competencies, the inaugural AbSPORU Graduate Studentship in POR (the ‘Studentship’) was launched in the summer of 2015.

The effectiveness of capacity development in health research is enhanced when it includes several interconnected components such as funding, education and mentorship [19]. The ‘Studentship’ incorporated education, networking and a variety of these related activities. The competition is conducted annually and available to first year masters or doctoral students enrolled in a health-related thesis degree at an Alberta post-secondary institution. Students receive funding and participate in capacity development activities that they can apply to their own graduate projects. Supervisor support is a required component of the competition application; however, supervisors are not directly involved in the capacity development activities or formal Studentship programme. The rationale for focusing on first-year students was to provide opportunities for the POR knowledge to be incorporated during the developmental stages of graduate projects. Additionally, few funding opportunities were available in the first year and the Studentship was intended to provide leverage for graduate students with a POR project to be competitive in other major competitions.

In 2015, 41 applications were received for the Studentship competition whereas 52 applications were received for the 2016 competition. Studentship awardees received a 1-year CAN$30,000 award and participated in capacity development activities provided by AbSPORU during the funding year (Table 2). There was no stipulation on the use of funds, provided that students were registered full-time, had demonstrated a POR approach in their graduate project and completed the required capacity development activities (Table 2). The primary objectives of these activities were to further Awardees’ knowledge of POR, develop practical skills for their own POR projects, and network with leaders and stakeholders in the POR community. Secondary objectives included “*to increase the quantity of POR at the graduate student level and as well as increase success in subsequent funding competitions*”; these objectives reflect the national SPOR mandate to increase the quality and quantity of POR [3].

**Table 2:** Studentship programme activities

Studentship programme activities	Description
POR training programme	Monthly modules (*n* = 8) with topics focused on each competency theme (Table 1). Sessions were held in person with local guest researchers/presenters and streamed to sites across the province
POR Summer Institute	Awardees attend this 3-day conference, which showcases keynote speakers, plenaries, workshops and networking; Awardees are provided an opportunity to give an oral presentation of their graduate project to develop public speaking skills and obtain advice/feedback from POR experts
Self-selected learning opportunities	- Shadowing POR committee or advisory meetings - One-on-one consultations with AbSPORU experts - Additional opportunities that arise through AbSPORU such as participating in working groups or assisting with a POR-relevant manuscript

*AbSPORU* Alberta SPOR SUPPORT Unit, *POR* patient-oriented research

### Competencies and the evaluation framework

The national CIHR and AbSPORU competencies in POR (Table 1) outlined the introductory knowledge and skills recommended for stakeholders interested in POR. The competency framework provided a guideline for the development of both content and potential outcomes in the Studentship programme. Competency themes were used as module topics in monthly workshops and sessions in the training programme. The Summer Institute conference, designed for a diverse audience, also used POR competencies to guide the content of workshops, activities and presentations.

Studentship programme outcomes were initially expected to be an extension of these competencies. For example, one learning objective in the general POR theme is: “*Participants are able to define and describe patient-oriented research*” [17]. While Studentship awardees demonstrated this competency, the overall outcomes of the impact narratives were far more powerful than responding only to the specific competencies.

Little is known about the outcomes and impacts of these types of programmes. Despite the importance of developing highly trained students and early career researchers, there has been minimal student follow-up in Canadian university departments, training organisations and research funders related to trainee outcomes [20]. While it may be easier to measure traditional outputs of trainee development, such as publications, successful funding applications and conference presentations, this may not represent the full picture in measuring impact [21]. Particularly for trainees’ development, measures such as publications and conference presentations may not be feasible at early stages in trainee programmes. A health research capacity development framework provided context for considering the awardee narrative reports through a non-traditional lens [21].

### Studentship capacity development activities

In addition to funding, each Studentship awardee was required to participate in the one-year training programme in order to meet the introductory competencies for POR. Monthly presentations and workshops were delivered in person and online. Topics included overviews of POR and patient engagement, which were then applied to specific research areas, including methods, data, clinical trials and knowledge translation. Presenters at the sessions included researchers and patients involved in POR in Alberta. The activities described in Table 2 highlight the capacity development opportunities designed for this programme. The programme evaluation explores the outcomes and impact of these activities on Studentship awardees, as research trainees, in the development of POR competencies.

## Methods – programme evaluation

### Data collection

The participants of the evaluation were the awardees of a funding competition that had its own criteria and peer-review process. The awardees were the population sample and their final programme reports provided the data for synthesis and analysis. A final report was completed by each awardee through online protected software managed by one of the funding partners. The final report included both quantitative and qualitative questions adapted from a narrative impact framework designed by the AbSPORU provincial funder to assess research impact. Rather than seeking to understand the impact of research, the adapted impact narrative was developed to better understand the impact of the Studentship programme and award funding on the progress and projects of the trainees. Awardees were asked to provide a narrative that addressed the impact of funding, training and opportunities provided in the programme. Additionally, they were to consider these opportunities in relation to their growth in POR, the development of their individual projects and the effect of the programme on their career goals or trajectory. The narrative impact guide directed awardees to consider outcomes as changes that occurred as a result of their experiences in the programme, such as increases in skills or knowledge. Impacts included how these changes influenced awardees’ projects or career trajectories. Additional information collected in the final report included traditional quantitative measures such as number of publications, presentations and additional funding awards received. Table 3 provides a summary of the questions from the narrative impact report form, highlighting the traditional report questions and the questions adapted from the narrative impact framework.

**Table 3:** Final report – funding impact narratives

Traditional report questions	Adapted narrative impact questions
Products of student work – publications, presentations	Describe goals for the term of the award, including what the student intended to accomplish as a result of the award and capacity development opportunities; describe the impact of the Studentship funding and activities in achieving these goals
Additional peer-reviewed awards received	Describe how the Studentship has influenced the student’s capacity to conduct, utilise and promote patient-oriented research, including new knowledge generated as a result of the funding and/or programme activities
Active collaborations or partnerships secured	Describe changes to the research project, career goals, or ability to participate or influence the research or external environment as a result of the outputs identified; illustrate any opportunities to promote or influence the developing culture of POR in Alberta
Capacity development activities completed	Describe the additional/optional activities engaged in during the Studentship programme, including the role you had in the activity and the result of the engagement to person, professional and/or research development

The provincial funder, as the Studentship administrator, collected awardee responses through a secured server and shared these with the authors via protected documents to assess the programme design, outcomes and impact of activities on the research projects as well as other outcomes. Awardees consented to information being collected for the purposes of programme evaluation, administration, promotion and publication at the time of award acceptance, which eliminated a separate consent process. All data were anonymous, with distinguishing characteristics of awardee experience and outcomes removed or minimised whenever possible.

### Analyses

The Studentship final report questions were structured in a way to group responses in pre-determined categories for synthesis; however, additional themes arose organically from the information, experiences and outcomes provided in the responses. MKR analysed the report data manually, extracting and grouping categories of the data that surpassed the structured report categories into additional themes and subthemes; MH and BKS reviewed these for completeness and consistency. These themes reflected the short-term outputs and impacts of the Studentship programme. Quantitative measures provided context and awardee characteristics. Descriptive statistical analysis was not conducted as the purpose of this programme evaluation was the narrative impact entries. The following sections identify the outcomes and explore the impact of the Studentship programme for individuals who completed this programme in 2015 and 2016. The results are discussed in relation to relevant literature in trainee and capacity development as well as a research capacity development framework [21].

## Results

Twenty-one awardee narrative reports were completed following the first 2 years of the programme. Awardee characteristics were described along with the main themes of the awardee reports that identified outcomes related to the development of relationships, increase in knowledge and skill, development of collaborations and partnerships, opportunity for mentorship and leadership development, impact on research project, career trajectory, and significance of the funding.

### Awardee characteristics

The results represent the 21 graduate students who were awarded a Studentship in 2015 and 2016; 9 awards were granted in the 2015 competition and 12 in the 2016 competition. The Studentship programme year follows the competition year (i.e. 2015 competition awardees participated in their programme from January to December 2016; 2016 competition awardees from January to December 2017).

All awardees were in their first year of a thesis-based health-related masters or doctoral programme in Alberta. Awardees were enrolled in one of three major universities in Alberta with a variety of departments and disciplines represented (Table 4) and a combination of both masters and doctoral students each year (Table 5).

**Table 4:** Distribution of awards to students representing disciplines/departments at Alberta-based universities

Awardee representation per discipline/department	Community Health Sciences/Health Sciences	Nursing	Public Health	Physical Therapy	Paediatrics	Radiology	Psychology	Nephrology
2015 Total = 9	3 (33.3%)	3 (33.3%)	1 (11.1%)	1 (11.1%)	1 (11.1%)			
2016 Total = 12	4 (38.4%)	0	1 (7.6%)	1 (7.6%)	1 (7.6%)	1 (7.6%)	3 (23.1%)	1 (7.6%)

**Table 5:** Awardee distribution across graduate degree programmes

Studentship year	Awardee degree programmes		
	Masters students	Doctoral students	Total
2015	2	7	9
2016	5	7	12

A number of traditional outcome measures were also collected in the final report in order to provide context for the narrative impacts and outcomes reported as well as offering some consistency with other award and capacity development programmes (Table 6). These measures are accurate only to the time of report submission and do not reflect any additional publications, presentations or funding secured after the close of the report period.

**Table 6:** Traditional measures of trainee development applied to Awardee final reports

Year (number of awardees)	Traditional outcome measures in trainee development		
	Published manuscripts	Conference presentations	Number of subsequent awards secured
2015 (9)	9	23	9
2016 (12)	20	31	7

## Final report analysis

Throughout the implementation of SPOR, both provincially and nationally, a primary focus has been on developing capacity as seen through achieving competencies. It was surprising to discover that the impacts were not only about knowledge and skills but also about relationships, changes in perspectives, and leadership development.

The principles of health research capacity development provided a framework to evaluate the success of capacity-building interventions at the individual, team, organisational and supra-organisational levels [21]. The individual level of health research capacity development evaluation is most relevant to this programme as it focuses exclusively on the training and development of individual students. This individual level is also described as a micro-level activity, focusing on building knowledge, skills and competencies [22]. In the research capacity development evaluation framework, the principles of capacity development at the individual level include the development of appropriate skills and confidence through training, supporting research that can be applied to practice, enhancing capacity through partnership and collaborations, appropriate dissemination, sustainability, and appropriate infrastructure [21]. These are summarised in Table 7. The following excerpts from awardee final report narratives echo many of the individual outcomes of capacity development represented in the framework [21].

**Table 7:** Summary from Cooke’s framework [21] for research capacity development at the individual level

Principles of research capacity development	Evidence at the individual level
1. Development of appropriate skills and confidence, through training and creating opportunities to apply skills	- Progressive skill development - Confidence building through sharing new skills with others, applying skills in new situations, working with other professional groups in research
2. Research capacity development should support research ‘close to practice’ for it to be useful	- Patient-centred outcomes as measures in projects and impact of project on patients’ quality of life - Critical thinking used in practice
3. Linkages, partnerships and collaborations enhance research capacity development	- Gaining and sharing knowledge - Increased number of partnerships and working inter-professionally
4. Ensure appropriate dissemination to maximise impact	- Papers in research and practice journals, conference presentations, applied dissemination of findings
5. Continuity and sustainability	- Successful access to funding, continued contacts with collaborators/linkages, continued supervision
6. Infrastructure	- Project management, mentorship and supervision structures

## Relationship building

Awardees consistently reported programme outcomes related to relationship building with stakeholders and others. This included networking, mentorship, collaboration and partnerships.

### Networking

Awardees were provided opportunities throughout the programme to connect with experts, peers, researchers and patients, particularly through the Training programme and Summer Institute conference. They described feeling “*fortunate to network with the presenters after their session”* (S02) and appreciating “*being able to network and meet new researchers and students interested in patient-oriented research over the course of the year”* (S06).

Networking also included opportunities to extend oneself beyond disciplinary boundaries, providing “*exposure to POR outside of my field and across disciplines”* (S17).

“*During the term of the reward there have been numerous opportunities to network. Every POR seminar included a group activity, which enabled me to discuss not only the seminar topic more thoroughly with individuals from other professions and backgrounds but also my research with them and vice versa. These discussions were always thought provoking and I enjoyed the opportunity to hear from individuals who viewed the topic from different angles due to their unique training.”* (S09)

Awardees also identified other advantages of networking opportunities, such as “*…interactions with others who share this belief* [in POR]*”* (S10) and learning first-hand from patients about “*...their perspectives and to hear how important the ability to participate and contribute is for them”* (S12).

Students were hopeful that the networks created during the term of the award would be of benefit as they progressed through their graduate programmes. These connections “*...have been invaluable and will be essential when establishing my research career at a later stage of my degree*” (S14).

### Mentorship

Aspects of the Studentship programme, such as interaction and discussion at events, contributed to developing formal and informal mentoring relationships:

“*These experiences have led to mentorship by professors and researchers that would have otherwise not been possible… through mentorships, additional resources have been provided such as access to new learning materials and introductions to researchers in the same field.”* (S09)

“[The] *significant impact of the personal mentorship and guidance … received from the relationships I have been able to leverage and build as a result of my studentship.”* (S01)

### Collaboration and partnerships

Collaboration and partnerships were sometimes developed as an extension of the networking activities that took place, particularly during events such as the Summer Institute or seminars:

“*Together with another student we developed the idea to create an innovative research priority setting strategy in our population of interest.* [This particular collaboration grew to include] *establishing partnerships within the core research team… and engagement with government and health organization leads.”* (S05)

The benefits of collaboration during the funding year included assistance with study recruitment, such as “*increased access to a population that fits the inclusion criteria for my research”* (S07) or influence on existing collaborations that were “*strengthened after receiving the award and as a result of this POR research project”* (S20).

## Increase in knowledge and skill

Awardee reports indicated that POR concepts and strategies were not only new to them at the beginning of the funding term but largely outside of traditional research education:

“*Despite my nursing training and background being based in a patient-oriented approach to care, the transfer of the philosophy into a research context was a challenge for me*. *Having been trained within traditional schools of thought regarding health research methods, many elements that are core to the POR approach were either unknown to me or were outright discouraged.”* (S01)

Awardees described increases in knowledge as demonstrated by a general understanding:

“*Participation in POR training sessions and seminars on patient-oriented research helped me to gain the understanding and language to explain what patient-oriented research is and why it is important.”* (S12)

A link from theory to application was also evident:

“*Learning how to actually engage patients at various stages of the research process and which research methodologies are most appropriate and lend themselves well to patient involvement.*” (S04)

Practical POR skills gained and reported by awardees included:

“*...How to build a strong patient-researcher team.”* (S13)

“*How to write a lay summary and why this is a critical skill for engaging patients.”* (S17)

“*Different ways of engaging patients at various stages throughout the research design process, including at the very beginning as stakeholders who can bring important contributions and perspectives forward, before study design even occurs.”* (S12)

Perhaps one of the most critical skills gained was in communication:

“*Understanding the* [POR] *terminology.”* (S19)

“*Developing the language to explain POR and why it is important.”* (S13)

“[The] *breadth of POR and POR vocabulary.”* (S21)

This introductory knowledge:

“[Allowed] *me to develop an in-depth understanding of the POR model.”* (S19)

“[Provided] *the confidence to implement more patient-oriented methods* [in their work].” (S08)

After completing the introductory sessions, some students pursued further knowledge on POR in order to apply it to their own work:

“*I also spent significant time doing my own research and reading about POR, and how to take these themes and apply them to my own research*.” (S03)

## Change in attitudes and perspectives

The changes in attitudes and perspectives about POR varied for awardees as this aspect is very individual and highly dependent upon previous notions and ideas as well as on the influence of previous careers, education and mentors. The types of growth shared in the awardee reports included broad indicators:

“*Being a funded SPOR student has allowed me to … reframe my thinking about my future career goals and POR.*” (S06)

“*Through the studentship I was able to gain a new perspective on what patient-oriented research really means: true engagement involves moving from the idea that the patient is a passive passenger to an active partner.”* (S02)

This was also evident in their self-reflection, which provided opportunities to re-evaluate their planned graduate research:

“*I examined my thesis proposal through a more critical lens* (to ask), *Why is this work important? How will it allow healthcare practitioners to provide better care to patients and how will it ultimately benefit patients?”* (S08)

Networking with peers, patients and researchers on POR topics ignited a passion for POR and motivation to focus on this type of research:

“[ I developed a] *passion to pursue patient-oriented research.”* (S14)

“[These interactions] *gave me an optimistic view towards where POR is headed in Alberta.”* (S15)

“[They] *encouraged me to think differently.*” (S20)

“[Participation in the programme] *gave me more cause to self-reflect and delve deeper into understanding the impact of health research to the public and its meaning as perceived by the patient.*” (S19)

## Leadership and initiative

The opportunities provided as part of the awardee programme have shown numerous positive results in the development of leadership qualities and opportunities for many of the awardees. Often, these opportunities were within the awardees’ peer group, such as “*attending the sessions allowed me to provide resources to fellow students and help them make their work more patient-oriented*” (S05) and being able to “*introduce other students, and consequently their supervisors, to SPOR and POR*” (S15).

Other leadership roles developed within awardees’ graduate programmes or faculties, such as “[I received] *increased invitations to networking events among researchers and graduate students, even guest lectures”* (S13). The awardee programme also “*raised their profiles within the faculty”* (S04).

Awardees also indicated that they had opportunities external to their own programmes and departments, including a number of conferences:

“*Taking part in the webinars has allowed me to gain skills in patient engagement research and qualitative research. These skills are sought-after within my research niche. As an example, I have been asked to lead the analysis of the qualitative data set that* [a research group] *has been collecting over the past few years. I will be able to publish 1–2 first author papers from this data set.* [The research group] *has also asked me to collaborate on two grants.”* (S02)

“*There was genuine curiosity regarding my work, as other researchers saw POR as an untapped yet much needed approach… from these conversations, I networked with leading scholars in my field who expressed an interest in collaborating with me to leverage my POR knowledge, both now and in the future.”* (S03)

In general, awardees found themselves in ambassador roles and were able to leverage numerous opportunities “*to promote POR with a variety of audiences at local, provincial, and national levels*” (S11). Two awardees, in particular, contributed to major initiatives inspired by their experience and what they learned in the Studentship programme:

“*The learning gained from the training and contacts made through this studentship helped me realize how often research and the needs of end-users are mismatched. It emphasized a major need for bedside-to-bench practices. As a result, I co-founded a student-led initiative… that seeks to accomplish a step in addressing this challenge. The initiative garnered immense support from the university and broader community.”* (S20)

“*With my passion for POR continuously on the rise and with my aim of keeping the spirits of POR with me as I advance in my career, I saw a gap in the scientific community for a Canadian POR journal. With this, I connected with* [a national journal] *for a potential collaboration, and established a POR collection. This collection will thrive* [through] *a POR Advisory Panel, which I will co-lead with a SPOR member. I am very fortunate to have pursued this idea and am very thrilled at how it has and continues to develop.*” (S10)

## Funding

Funding competitions for early-stage graduate students are limited and the Studentship programme is designed to fill this need. The expected benefits of this early funding included being able to focus on their studies and participate in additional training opportunities:

“[The award] *allowed me to focus approximately 90% of my time on my research activities.”* (S06)

“[I could] *attend additional training and conferences.”* (S12)

“*Work fewer hours.”* (S14)

“*Not having to worry about the financial aspects of being a student.”* (S21)

A secondary objective of the programme is to assist awardees to demonstrate strong research projects that would be more likely to succeed in subsequent health research funding competitions. This is also one reason why the award is designed as a 1-year programme to act as a catalyst for additional project funding, which is typically in the fall of each year. Many factors influence the success of funding applications; therefore, it is not possible to directly connect any subsequent funding success to this programme. However, a number of awardees expressed their feelings about subsequent funding in their final reports:

“*The experience gained from applying for the SPOR Studentship was leveraged to apply for and receive a grant…”* (S09)

“*I have no doubt that my SPOR Studentship contributed to my success in other scholarship competitions… these scholarships provided critical financial support that allowed me to focus exclusively on my academic, research, and clinical endeavors...”* (S16)

“*I believe that my participation in SPOR demonstrates my eagerness to conduct health research that is meaningful to the patient. In turn, I think that this benefited my other applications and made me more competitive.”* (S19)

## Project and career impact

The timing and design of the Studentship programme is intended to provide awardees with the opportunity to incorporate POR strategies and tools learned in the programme to conduct their own projects. While acceptance to the programme indicates that the projects already demonstrated some POR focus or approach, the reports indicated that many students made subsequent changes and amendments to their proposed study based on their learning and experience in the Studentship programme.

Awardees made project changes as a result of their participation in the programme. These changes reflected their newly acquired knowledge of POR:

“*Methodological in nature and reflect a desire to place the perspectives and concerns of patients into the instruments we will use.”* (S01)

“*Actually re-designed my PhD project to include a true patient engagement piece.”* (S02)

“*Convened a patient engagement panel before I finalized my interview guides and initiated data collection.”* (S10)

“*Restructured the interview guide to include questions about patient research priorities.”* (S05)

“[Developed] *an enriched lit review on research with patient engagement… changing my approach with a theoretical framework with the knowledge foundation on POR.”* (S13)

In the long-term development of career building, it is more difficult to demonstrate cause and effect; however, many awardees indicated a shift in career goals, trajectory or opportunities after participation in the Studentship programme. Some plans involved further graduate studies or a career in academia:

“*My career goals have significantly changed since the start of my SPOR studentship… I have decided to continue on into PhD… this decision is the result of the conviction I have developed to tackle challenging health problems that are of importance to patients.”* (S01)

“*At a broader research level, my end goal post-PhD is a career in academia... each academic has his or her own niche, and I have started to see mine founded on POR.”* (S10)

## Discussion

Awardees completed a 1-year training programme that incorporated elements of capacity development, including funding, education, networking and mentorship. Outcomes were explored through qualitative data collected during the programme evaluation. Most awardees reported a positive impact at some level, whether it was a simple increase in knowledge or a profound change in perspective. For most awardees, any training in POR was a new opportunity as current graduate programmes in Alberta do not include courses or content on POR as part of the curriculum. While applications to the funding competition were required to demonstrate some aspect of POR, awardees consistently reported that they gained knowledge and explored novel ideas that were enhanced by opportunities to connect with experts and patients sharing real-world examples of POR.

The status of being granted a funding award, along with training in a current focus area of health research, afforded organic opportunities in leadership development for many awardees. They felt fortunate to find themselves in unique positions within their peer groups, faculties or the broader research environment due to the exclusive opportunities provided in the programme. While being awarded any funding can assist in building a student’s profile, the development of first-year students emerging as knowledgeable trainees in POR was a relatively unforeseen outcome. It was expected that some awardees would move forward as future leaders in their research areas; however, opportunities to take on more prominent roles as graduate students indicated both genuine interest in POR and a realisation of its potential.

In a broader sense, capacity development interventions are thought to function as a mechanism that releases potential energy from an individual and provides an outlet for motivation and creativity [19]. This points to additional unanticipated outcomes where a few of the highly motivated and innovative awardees pursued ideas generated through programme experiences. They went on to implement these initiatives with widespread success. While these outcomes are entirely the result of awardee initiative, the impact stories shared in the final reports indicated inspiration from attending Studentship events and programming. Other key catalysts were the discussions, dialogue and networking that took place with a variety of stakeholders during programme activities.

The outcomes described above demonstrate impact occurred beyond achieving the initial competencies that guided programme design. For this reason, the Cooke [21] framework for evaluating research capacity development was utilised to further explore these impacts through a different lens.

### Awardee outcomes and the research capacity development evaluation framework

Due to the unique nature of this training and funding programme, comparisons with the literature are limited. Follow-up with students post-award is rare and there are no formal evaluations in the literature of combined funding and development programmes in Canada [16]. Therefore, the Studentship awardee outcomes were re-examined using the principles of health research capacity development outlined by Cooke [21] to contribute to programme evaluation as well as to provide considerations for future programme development.

#### Principle 1. Development of skills and confidence through training

Numerous awardees reported increased knowledge and skills. The new knowledge included understanding concepts, terminology, and awareness of key strategies and considerations for POR. One of the defining characteristics of this base knowledge in POR is the reported ability to communicate effectively about POR, to use appropriate language and terminology and, in doing so, confidently enter into dialogue with a multitude of stakeholders. This was an expected outcome directly aligned with the programme goals and the framework.

The increase in confidence expressed by several awardees was an encouraging outcome. As first year graduate students, embarking on an approach to health research that was likely different from other students in their programme, is a formidable challenge. Yet, as indicated by the leadership development opportunities described above, this challenge and distinguishing characteristics of their projects were embraced, resulting in numerous stories of emerging confidence. While not an explicit goal of the programme, this confidence in POR as a research approach, and in one’s own ability to engage, communicate and advocate, enhanced other aspects of the programme such as the ability to maximise networking opportunities and build strong relationships, collaborations and partnerships.

#### Principle 2. Supporting research ‘close to practice’ for it to be useful

The nature of Awardee projects and specific topic areas were not under the purview of this programme. Graduate students work with their supervisor on project selection and its relationship to practice as part of their overall graduate programme. However, this principle is reflected in the awardee outcomes as evidenced by the project changes that were made. A key tenet of POR is to provide research that is useful in clinical practice and relevant to patients; the numerous changes made by awardees to demonstrate not only the integration of POR but the priorities and contributions of patients is an indication of commitment to and the potential usefulness of their project to practice.

#### Principle 3. Linkages, partnerships and collaborations

Networking and collaborations were defined as activities that support people to work together and as a result improve knowledge, processes, or increase outputs [19]. The Awardee outcomes showed a significant impact related to networking opportunities, connections made with multidisciplinary stakeholders as well as development of partnerships and collaborations.

Mentoring is a key component of capacity development in health research, particularly for trainees [10, 23]. Mentoring is also acknowledged to provide access to a community of like-minded peers and colleagues while also providing a place of belonging [24]. With the relative novelty of POR, particularly within the graduate student population, it was imperative to provide access to the larger community of POR experts within the province to create additional supports and networks for the Awardees. The design of the programme’s mentorship section took on an organic, opportunity-driven approach as deliberate or pre-determined mentorship relationships may have overlapped with supervisory roles already in place in awardees’ graduate programmes. Instead, the programme design aimed to offer voluntary opportunities that awardees self-selected based on their needs, interests and relevant activities to their project and development.

Capacity development is required to develop research collaborations through meaningful and effective communication [5]. Many Awardees noted the importance of education as well as learning the foundations of POR in order to feel confident and be able to network and engage in effective discussion with POR experts and stakeholders. The interconnected activities of education and networking filled a critical need for awardees by providing them with the ability to connect with others as well as the context where these connections took place. Some awardees took initiative beyond the opportunities provided and sought out additional avenues for networking among their departments, disciplines or within their target population as they pursued engagement practices. Awardees demonstrated growth and development beyond the designated Studentship activities through maximising opportunities to give presentations, join advisory committees, be invited to research teams or provide mentorship to peers.

#### Principle 4. Ensure appropriate dissemination to maximise impact

The 1-year timeline of this programme precludes reporting on many capacity development outcomes that often present in mid- or long-term timeframes. However, the competencies developed by AbSPORU (Table 1) include a knowledge synthesis and knowledge translation component to ensure that awardees are provided fundamental knowledge in these key areas. Awardees noted that their consultations with members of the AbSPORU Knowledge Translation Platform were important to develop the knowledge and skills needed to disseminate their work and create this plan early in study design. Associated skills, such as communication, language and terminology, and lay language approaches were also flagged as useful topics in the Studentship programme that awardees incorporated into their ability to disseminate information to a variety of stakeholders.

The number of publications and presentations are traditional outcome measures in academia (Table 5). Awardee narratives highlighted additional opportunities to participate in departmental presentations, advisory committees and other leadership roles to disseminate both their individual projects as well as information about POR in general and its role in health research.

#### Principle 5. Continuity and sustainability

Access to funding is emphasised in the evaluation framework [1] as an integral part of sustainability in capacity development and allows trainees to devote their efforts to their research and training [13]. Traditional outcome measures (Table 6) demonstrated that awardees had secured funding at the time of report submission, with additional awards received since SPOR Studentship programme completion. A cautionary stance should be taken when assuming a cause and effect relationship between programme and funding. Despite the positive impact reported in successful grant productivity after mentorship and training [24], the difficulty in attributing funding success to the Studentship programme alone remains as external factors might have also created positive or negative impacts [19]. The success of awardees in subsequent funding competitions nonetheless indicates a growing, continued support of POR projects and the individual students as they continue in their programmes and careers.

Continued success of POR projects with mainstream funding competitions was a goal of the Studentship programme in an effort to build the momentum of POR in the broader health research community. It may still be years before the trend of POR success in funding can be evaluated for awardees of the programme, yet awardees have made connections in attributing further success to skills, confidence and experience gained as part of the Studentship programme.

Often, the aims of capacity development projects are designed to improve practices that will then be maintained moving forward [15]. In this case, sustainability of POR as an approach in health research is also related to outcome measurement. First year trainees were selected as the target audience for this initiative, which aimed to support the future of POR in Alberta. While the funding and support described in this paper was only provided for a limited period of 1 year, the goal is for the practices of POR to be maintained by trainees as they become key stakeholders and sustain this approach in the research community.

#### Principle 6. Infrastructure

Two types of infrastructure were identified as paramount in the development of graduate students in POR – on an individual, supervisory and graduate programme level as well as provincially at the funding level. A strong supervisory and mentorship team was a critical part of the application to the funding competition. To be successful, it was imperative that supervisors were involved in and approved the applicants’ POR projects to ensure continued support of the POR project as they continued on from the Studentship programme and completed their graduate projects.

Contextual factors are a significant consideration in the infrastructure available to each student as graduate programmes, supervisory relationships, research environments as well as departments and disciplines can vary in the specific infrastructure available to each student. While these contextual nuances may vary widely, graduate programmes in Alberta are competitive with high calibre faculty and programme requirements at each institution, which suggests that suitable infrastructure is built into the graduate programmes.

Adequate support structures and training are a key component to ensure trainees can incorporate best practices in current and future work [11]. As an organisation, AbSPORU played an important role in the infrastructure and support provided to students both in their funding year and in longer-term relationships maintained post-award. The supports, services, consultations and mentorship provided by AbSPORU and others in the provincial POR community were also critical factors in the success of the Studentship programme. This is clearly illustrated by the numerous awardee reflections that demonstrate the profound effect on all aspects of their development that included individual learning, perspective, project design and career goals.

The POR competencies were the driver for the Studentship competition and programme development. However, the health research capacity development framework [21] identified outcomes that extended beyond knowledge and skill acquisition and provided a basis for developing additional capacity in areas such as confidence and collaboration. The evaluation framework [21] addresses critical aspects of trainee experience, beyond basic learning objectives, to incorporate environment, support and relationship building.

Despite the comprehensive use of competency and evaluation frameworks, personal development in traits like leadership and initiative as well as individual shifts in perception is yet unaccounted for. In the research trainee population, one could argue that these abilities may be as essential to development as those identified in existing literature and models. Increases in confidence and formidable initiative demonstrated by awardees may indicate that there are additional dimensions of capacity development yet to be included in evaluation frameworks or models.

### Moving forward: the trainee role in a POR culture change

In a scoping review of POR competencies in the literature, Mallidou et al. [5] described POR as a paradigm shift in the health research environment. Developing capacity in POR is a critical step in supporting this culture change, as patients, public and community are moving into new engaged roles in health research – more than historically just as subjects. Rouleau et al. [4] emphasised the importance of preparing the next generation of researchers for work in POR, particularly when transformative work lay ahead in the ongoing evolution of this approach.

The importance of ensuring that graduate students are prepared for a change and shift in the health research landscape may help to bridge the gap in what is currently offered in graduate training at post-secondary institutions [25]. Capacity development requires additional education, training, tools and resources beyond the traditional infrastructure. The awardee reports illustrated impact in all areas and detailed how training, tools and resources created change in their projects, skills and professional development. In addition to preparation for future careers, other benefits are often derived from graduate student audiences in multidisciplinary capacity development as they bring enthusiasm and new ideas [25].

This study provides new knowledge about the implications of funding and capacity development activities for POR trainees in Alberta. These results suggest funding provides the incentive, motivation and practical support needed to pursue additional training beyond the formal courses required in graduate programmes. The capacity development activities provided through the Studentship initiative resulted in a multitude of benefits, including increased knowledge, changes in perspective, refined POR projects and inspiration for new career goals. Evaluation methods for trainee programmes are often associated with traditional measures of academic growth such as publications or gaining additional funding. Due to the unique nature of this programme as extra-curricular in nature and focused on a new approach to traditional research, a strategic decision was made to focus on different kinds of evaluative questions. The goal was not to demonstrate empirical measures for increases in knowledge or competency but to generate narrative data that explores broader individual impacts which may, over time, contribute to a change in culture through the creation of a community of trainees and early career researchers who value POR and the principles it represents.

Further research is needed to determine how the competencies developed by SPOR and AbSPORU are associated with learning needs of health research trainees and future job requirements. In identifying expected outcomes for future programme planning, it is also important to consider maturation of the SPOR initiative itself. This creates a need to reconcile early identification of competencies where capacity was low and POR was a new concept with a more sophisticated learner population and provincial context as we move into the fifth year of the Studentship programme.

The focus of this evaluation was capacity at the individual level, yet attention is also required in the broader system in which individuals work, study and contribute for capacity development to read optimal implementation and sustainability [21, 22]. Long-term assessment beyond the individual level may also provide an essential look at broader impacts or trickle-down effects of focusing funding and capacity development efforts at this level of trainee, including impacts on their research groups, departments or networks.

The infrastructure of AbSPORU as well as the universities in which AbSPORU operates and awardees complete their academic programmes are integral to the development of the trainees. Similarly, without growth and development at the organisation and supra-organisation levels [21], or the meso- and macro-levels [22], there would be little structure to enable these trainees to continue moving forward in their POR work, as trainees and as future researchers.

The sustainability of the Studentship competition and programme in general are also inextricably linked to the infrastructure of the health research ecosystem as well as the funding structures and timelines of the SPOR initiative. The capacity development activities were implemented at little cost with collaborations and partnerships across the research universities in Alberta; however, funding for the awards is dependent on the future of the SPOR initiative, both nationally and provincially. While aspects of the capacity development activities, such as education and training opportunities, could be adapted for integration into other programmes or existing structures within academia or trainee development, the award competition administration and funding may be time-limited to current SPOR grant funding.

## Limitations

Based on the nature of this programme, there are no control groups to compare results or similar programmes for first year graduate students in Alberta. While matching cases of awardees with students of similar backgrounds and funding potential, there are too many potential confounding factors to estimate what awardees may have accomplished without this programme. High-achieving individuals may have developed beneficial partnerships, collaborated with experts in their fields, secured subsequent funding and numerous other achievements without the Studentship funding or programme. However, the specific outcomes demonstrated here, particularly with the focus on POR, were less likely to occur without the support of this programme and the variety of opportunities afforded to Awardees.

The self-reporting nature of the data collection may be a limitation and no additional data exists to triangulate survey results. This data was utilised as an important first step in understanding the short-term outcomes of the funding and capacity development activities of the programme. In addition to the potential self-reporting bias, awardees may have exhibited a positive bias towards the programme based on the funding received.

Finally, AbSPORU staff members conducted the evaluation, which may have introduced a bias towards the positive results of Studentship awardees. To minimise this bias, one person (BKS) distributed and collected the evaluation data, and two other team members analysed the data (MKR, MH).

## Next steps

Two aspects of moving forward are worth considering – the role of generic versus role-specific competencies and the value of developing content versus assessing outcomes guided by different frameworks. A lack of comprehensive and consistent competencies for stakeholders in POR has been identified [5]. Given more is known about outcomes and learner impacts, additional research is needed to determine how effective it is to develop content based on competencies. Identifying a hierarchy of competencies specific to stakeholder groups or roles in health research could be undertaken. These stakeholder group competencies could then inform educational design and support systems for a range of POR knowledge, skills and leadership in all areas of the health research landscape.

The competency framework was useful in guiding content for capacity development activities; however, in this exercise, it falls short of appropriate utility in programme evaluation. For future iterations of the programme, while trainee development may continue to be informed by general competencies, evaluation of outcomes and impact, particularly beyond knowledge and skills to include interpersonal relationships, collaboration and leadership, need to be informed by a more comprehensive framework. Valuable outcomes may be left undocumented if these additional impacts are not consistently evaluated and reported.

In the continued development of novel programmes, such as the AbSPORU Studentship, it may be worth considering identifying competencies from multiple sources – general competencies identified by the national SPOR programme as well as using results and feedback gained from the final report narratives. A comprehensive competency framework that acknowledges the acquisition of these additional characteristics may then inform subsequent iterations of the programme. Further exploration of capacity development in other fields may also provide insights and lessons learned to continue to establish and improve robust training programmes for trainees and other stakeholders in POR.

The 1-year Studentship programme did not provide opportunity for multiple reporting or feedback opportunities. Multi-year programmes may benefit from tracking progress longitudinally; however, this was not feasible given the short time-span of the programme. The AbSPORU Studentship programme is relatively new, although students in the early cohorts are now graduating from their respective academic programmes; interviews and surveys are planned for these students to identify longer-term outcomes of awardee development. Longer-term tracking of trainee progress and impact (over 3–5 years) may prove challenging, particularly to determine what can be directly or indirectly attributed to the Studentship programme. In support of programme sustainability, tracking career trajectories and outputs of research activities will serve to justify spending and encourage continued funding [20]. Considering SPOR and AbSPORU are still in the first phase of operations in Canada, the importance of tracking and reporting these outcomes and the impact of trainee development should not be underestimated.

## Conclusion

The AbSPORU Studentship programme facilitated capacity development for graduate trainees in POR during a 1-year funding and training programme. The outcomes reported included the development of personal competencies, leadership capabilities and opportunities, collaborations and partnerships, new perspectives on POR and health research as well as changes in project design or career goals to reflect priorities in POR.

Early results indicate that programme objectives have been met; additional impacts reported by awardees indicate the need for a revision of the guiding competencies to reflect the breadth of outcomes demonstrated. While specific learning activities may be adequately designed and evaluated using the competency framework, the use of the health research capacity development framework [21] provides a more comprehensive evaluation of awardee outcomes that encompass a greater scope of development. Some outcomes are still unknown, such as career trajectories and research outcomes, and will be further evaluated as awardees complete their graduate programmes and move forward in their careers.

## Data Availability

The data used in this evaluation are available from the corresponding author on reasonable request.

## Abbreviations

POR: Patient-oriented research
SPOR: Strategy for Patient-Oriented Research
AbSPORU: Alberta SPOR SUPPORT Unit
SUPPORT: Support for People and Patient-Oriented Research and Trials
CIHR: Canadian Institutes of Health Research
